# Healthcare Professional Presenteeism during a COVID-19 Outbreak in an Australian Rural Healthcare Environment: A Case Analysis

**DOI:** 10.3390/ijerph18168336

**Published:** 2021-08-06

**Authors:** Kathleen Tori, Thi Thuy Ha Dinh, Carey Mather

**Affiliations:** School of Nursing, College of Health and Medicine, University of Tasmania, Launceston 7250, Australia; thithuyha.dinh@utas.edu.au (T.T.H.D.); Carey.Mather@utas.edu.au (C.M.)

**Keywords:** presenteeism, health, rural, workforce, recruitment, retention, community

## Abstract

The recruitment and retention of health professionals in rural Australia is well documented. The COVID-19 pandemic has further exposed the precariousness of human healthcare resources within small rural communities. The external disaster of the COVID-19 outbreak described in this case analysis exacerbated the frail balance of sustaining adequate staffing levels and skill mix, which exposed behaviours of presenteeism within rural healthcare teams. An analysis of the complex of factors that led to the first nosocomial outbreak of COVID-19 within a healthcare environment in Australia demonstrates how rural healthcare environments are ill-equipped to meet the demands of unexpected external disasters. Using the Haddon Matrix to examine the factors that led to this outbreak provides us with the opportunity to learn from the case analysis. Health professional presenteeism contributed to the North West Tasmania COVID-19 outbreak and affected the hospital and health service provision within the region. Recommendations to mitigate risk for future disaster planning in rural healthcare environments include improved infection control strategies and a whole-community approach.

## 1. Introduction

The novel coronavirus (COVID-19) is a highly contagious infectious disease that has become a pandemic, with a global impact similar to the H1N1 influenza (Spanish flu) pandemic in 1918 [1]. The first cases of COVID-19 were identified in Wuhan, China in December 2019 and quickly spread to other countries. The exact origin of COVID-19 is being investigated, although according to Chen [2] (2020), Wuhan, China appeared to be the locality of initial exposure, with 42,638 confirmed cases of COVID-19, and over 1000 subsequent deaths, in the first three months. COVID-19 quickly affected over 32 provinces in China, becoming a worldwide public health issue as the virus spread to other continents [2]. The current COVID-19 pandemic has already caused over 3,530,837 deaths and over 169 million confirmed cases [3] (updated 1 June 2021), although the statistics continue to rise in the most affected regions across the globe. The United States of America, Brazil and India have experienced the highest death tolls, whereas Hungary and Czechia [4] have had the highest deaths per one million population and were amongst the worst-affected countries [4] (updated 1 June 2021). The subsequent emergence of more contagious virus variants has added complexity to managing the current pandemic. In planning the initial COVID-19 response, many countries implemented preventative measures in accordance with frequently updated advice regarding contagion transmission pathways and symptomology; measures which were also informed by lessons learned in previous pandemics [5]. Preventative public health strategies have included physical distancing, use of personal protective equipment (PPE) such as masks and hand sanitizers, rapid testing of suspected cases, extensive exposure tracing, quarantine of positive cases, and, more recently, mass vaccination programs, which have proven their collective effectiveness in mitigating and controlling the extent of COVID-19 [1].

Initially, the uncertainty regarding the transmission mechanisms of COVID-19, as well as the general unpreparedness and attitudes of the general populace and authorities [1], slowed the necessary responsiveness of preventative health systems. Added to a lack of understanding regarding the transmissibility, the implementation of differing strategies for different countries impinged on the ability to gain rapid and definitive control of the escalating situation [1,2]. The consequences, together with other contributors, particularly the premature relaxing of preventative measures, led to the overwhelming and collapse of affected health systems, especially those that were not adequately equipped or prepared for a large influx of patients requiring healthcare for this infectious disease. For example, the current COVID-19 crisis in India has revealed differing levels of preparedness and has disadvantaged already vulnerable populations [6]. The response to the pandemic in India has been hindered by inadequate public health such as hygiene and sanitation. An insufficient healthcare workforce, especially in the public health care sector, compounded by depleted or non-existent medical supplies, including PPE, hospital beds and oxygen delivery systems, as well as inadequate testing, vaccination, staffing and facilities has contributed to inadequate disaster management [6,7].

Person-to-person transmission of the disease in communities has been obvious; however, there were unusual cross-infections from healthcare providers as virus “carriers” to patients for whom they cared, and vice versa. This transmission has been attributed to employees working while unwell, which is more formally known as ‘presenteeism’ [8]. The behaviour of presenteeism is not new and was identified in pre-pandemic times in high presence-demanding industries, such as health care environments, factories or amongst school educators [9]. Presenteeism is more commonly a trait exhibited by women, older employees, and those persons with conscientious personalities [10]. Health care workers, mainly medical practitioners, and nurses, exhibit higher presenteeism behaviours than other professional groups [11], and particularly in underserved geographical rural and remote areas, where there is a deficit of alternate staffing. During COVID-19, presenteeism has become problematic when infected healthcare professionals care for multiple patients, often in more than one healthcare environment, while being unknowingly infectious, or waiting for COVID-19 testing results, effectively potentiating the transmission of COVID-19 [12]. Presenteeism was also found to be a contributing factor to the occupational acquisition of COVID-19 in industries such as abattoir workers [9].

Presenteeism is a behaviour in which employees attend their place of employment while unwell, due to an acute illness, or other medical or psychological condition [13,14]. The prevalence of presenteeism is identified as a relatively common behaviour within the health industry, estimated as being as high as 80% [15], and was a major contributor to the spread of COVID-19 in health facilities within Australia [9]. Occupational infection occurred in several healthcare facilities, most notably the North West region of Tasmania, as well as in residential aged care facilities (RACF) in Victoria and Queensland, during the recent pandemic where nursing staff continued to practice, at times across multiple sites, while diagnosed as being COVID-19 positive [9].

Contextually, presenteeism not only adversely affects healthcare professionals, and the well-being of professional colleagues; it may also impact negatively on patient outcomes [16]. Research has shown that working while at less than optimal health levels can lead to decreased mental acuity, increased medication/procedural errors, missed nursing care, increased patient falls [10,17], and can be postulated to have occurred during the COVID-19 pandemic, leading to heightened risk of transmitting the disease to an already ill patient [12,18], Rainbow et al. (2017) attributed presenteeism to a ‘self-sacrifice culture’ which leads healthcare professionals to continue to work while actively unwell and expect their colleagues to do the same, to avoid low staffing or inadequate patient ratios or skill mix. This seemingly acceptable behaviour is particularly problematic in geographically rural or isolated areas where the health workforce numbers are already maldistributed and many healthcare professionals work across multiple sites, such as in rural health facilities such as district hospitals and concurrently in RACFs [19].

Rural health facilities face substantial challenges in the recruitment and retention of an appropriately and adequately skilled health professional workforce, due to a lack of sustainable infrastructure and declining funding [20]. When exposed to a global pandemic, already stretched resources become even more dire, while trying to meet the health needs of small communities in a timely and accessible manner. Given the limitations, particularly in maintaining essential staffing, rural health facilities are prone to the behaviour of presenteeism, as the health professionals are reluctant to let their fellow colleagues down [9,13,16,20]. The complex of factors that led to the first local outbreak of COVID-19 within a healthcare environment in Australia are analysed.

## 2. Method

A case analysis was undertaken of the first nosocomial COVID-19 outbreak in North West Tasmania, Australia, which occurred in early April 2020. It occurred in both hospital and health service provision within the region. Data included details of the initial outbreak and progression incidence mortality, and organizational and government responses were predominantly sourced from two published reports—the interim report by the Tasmanian Department of Health [21] and an independent report [19] by Melick (2020). Additionally, data used to inform this analysis were sourced from government COVID-19 websites and news reports, with supporting literature for background information sourced from scholarly databases.

The Haddon matrix [22] was employed to provide a framework for investigating the complexity of factors that contributed to the outbreak of COVID-19 within this rural region. The Haddon matrix describes four overarching factors related to host (healthcare professionals), infrastructure, physical, and social environment, which contributed to the outbreak at two occasions of pre- and during the event, and how changes have been made or suggested in these dimensions post-event. The Haddon matrix is a model that was developed in the field of injury prevention to reduce morbidity and mortality by standardizing safety analysis (Haddon, 1968). Previous studies have used the Haddon matrix to explore preparedness during viral outbreaks such as severe acute respiratory syndrome (SARS) [23], flu [24] and Ebola [25]. The matrix, by analysing this local outbreak into its dimension of time and contributing factors, can be utilized as a practical tool to help understand the causes and to suggest improvements for future disaster planning.

## 3. Results

### 3.1. Case Study

#### Region and Development of Outbreak

The first Australian nosocomial outbreak of COVID-19 occurred at the North West Regional Hospital on the island state of Tasmania, Australia. The outbreak affected all health services in the North West of the island, including three hospitals and other health services [19]. The main locus of infection was at the North West Regional Hospital, which is 145-bed acute public hospital based in Burnie, Tasmania. This facility is co-located with the North West Private Hospital, which is a 48-bed health care service. Both facilities offer medical and surgical services, while the public hospital provides emergency services, the private hospital offers the only birthing service on the northwest coast of the island. Southeast of Burnie is the Mersey Community Hospital, which services the surrounding agricultural area, and jointly shares staff, the provision of stores and equipment with the North West Regional Hospital. There were a number of single rooms at the healthcare facilities; however, none were negatively pressured, or had anterooms for donning and doffing of PPE [19].

The timeline for the development of the outbreak begin with the first in-patient admitted to the North West Regional Hospital on 20 March 2020, followed by a second in-patient on 26 March 2020. On 3 April 2020, there were two positive COVID-19 tests of North West Regional Hospital healthcare professionals, a third staff member was reported as being COVID-19-positive on 4 April 2020 [19].

**Figure 1:**
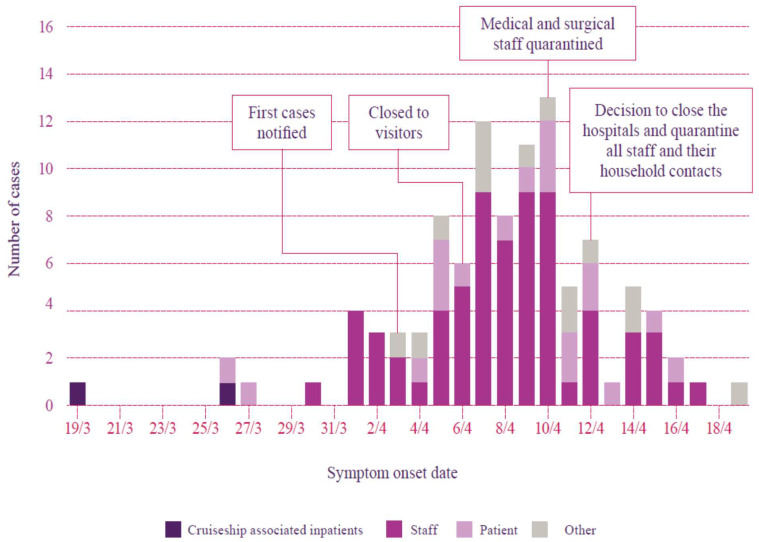
Case of COVID-19 associated with the North West outbreak, by date of symptom onset. Date & Month notated within the X-Axis for example 19/3 correlates with 19 March; 21/3 21 March etc. [19]: p. 24.

Figure 1 shows the COVID-19 cases associated with the North West Tasmania outbreak. The figure demonstrates that some healthcare professionals were already infected before the outbreak was announced [19]. Individuals were infectious for up to 48 h, with viral shedding prior to showing symptoms, and so it is likely that transmission of COVID-19 occurred inside the healthcare environment, and within the community, before staff realized they were infectious. This first nosocomial outbreak in Australia resulted in 138 cases, of which 80 were staff, 25 were patients, including one resident from a RACF, and 33 others, including close contacts of healthcare professionals. There were 10 deaths, of which nine were nosocomially acquired within the rural healthcare environment, and one resident at an RACF. There are two remaining deaths that are yet to be determined by the State Coroner [19].

### 3.2. Haddon Matrix Analysis

The Haddon matrix describes which cofactors contributed to the onset and progress of the local outbreak in North West Regional Hospital, including host (healthcare professional), resources, physical, and social environment on the horizontal axis and pre-, during and post-event on the vertical axis. [21]. In this case study, the host refers to healthcare professionals affected by the transmission of COVID-19 virus. Resources refers to the preparedness, provision, and usage of resources, for example PPE, to respond to the COVID-19 outbreak. The physical characteristics of the environment include the geographic location and design of healthcare facilities. The social environment encompasses cultural factors within the environment, such as legal and social norms. The dimensions of the matrix enable the identification of interventions that assist in addressing the issue being analysed [22].

The findings from the interim report [21] indicated that the physical environment impacted on the transmission of COVID-19 among staff and patients. There was movement of staff between facilities, including between the public and private hospital and the other health care facilities within the region. Additionally, the arrangement of corridors and rooms created opportunities for cross infection. Clinical handovers, ward rounds, meal breaks, management and staff attendance at committee meetings also contributed to the transmission of COVID-19 among the nursing, medical and other staff. The difficulty in maintaining social distancing and the capacity to maintain best practice in infection control procedures also contributed to the transmission of COVID-19 (Table 1).

The report also indicated there were a number of drivers for presenteeism among the health care workers employed at the North West Regional Hospital [21]. It was reported that staff presented for work because they did not want to ‘let colleagues down’ [21] (p. 24), due to fear of retribution for not showing up at work, due to misdiagnosis of signs and symptoms of COVID-19, due to underestimating the seriousness of the consequences of presenting for work while infectious, or due to concerns over resource constraints [21]. A lack of clear and consistent processes for the tracing and management of contacts for patients, staff, and the community were highlighted in the report [21]. Additionally, the continued reliance on paper systems within the health care service hindered the timely management of interrogating rosters or staff and patient movements throughout the health service, contributing to the transmission of COVID-19 within the North West coast of Tasmania [21]. The Haddon matrix before the event shows how the complex of host, resources, physical and social environments led to the COVID-19 outbreak.

## 4. Discussion

While COVID-19 created unprecedented public health concerns globally [26], the repercussions in a small Australian rural community that was unprepared and under resourced were multifactorial. Investigations into the outbreak in the rural health facility identified multiple contributors that exacerbated the outbreak of COVID-19; however, presenteeism of the healthcare workforce is the focus of this discussion [19]. As shown by the Haddon matrix analysis (Table 1), presenteeism was identified as a major contributing factor in both the interim [21] and independent reviews [19] of the COVID-19 outbreak. Twenty percent of health professionals continued to go to work while exhibiting COVID-19 symptoms. The Haddon matrix [22] analysis highlights that during an event, healthcare workforce presenteeism impacts situations where resources are already compromised [9,13,16]. If healthcare professionals continue to attend their place of employment while unwell [27], they pose a risk to colleagues, patients, and members of the community external to the place of employment. This behaviour can also contribute to localized outbreaks that potentially have major economically and socially disruptive consequences, not to mention heightened community anxiety, which occurred in this case [9,28]. Additionally, healthcare professionals working across multiple sites, as was identified for this rural health facility, effectively provided carriage of COVID-19 into RACFs [19].

Given that the nursing constitutes a large proportion of the health care workforce [29] and provides direct patient care, it is unsurprising that nursing staff, as well as medical and allied health professionals, were implicated as contributing to the presenteeism in the North West outbreak. Research has identified nursing as an occupational professional group with high presenteeism behaviours [13,17,30]. A longitudinal study [30] of 21,000 nurses identified that on any given day, a large percentage of the nursing workforce report to work while their health status is less than optimal. Physical, psychological, and other variables, such as domestic, child or elder care issues, that affect presence and productivity can adversely affect both caregivers and patient safety [17].

There are factors why nurses attend work while being physically or psychologically unwell. Fear of reprisal from employees for failing to attend designated shifts, feelings of letting colleagues down in an already understaffed situation, the necessity of earning an income, having a strong work ethic and not recognizing the severity of illness were identified [19]. These findings are consistent with previous research on presenteeism; several authors [18,31] purported that the culture of the healthcare industry perpetuates this behaviour. From a hospital or healthcare facility perspective, it has been found that there is a culture of expected presence or a need to be seen, this being a particularly strong feature in the Asian healthcare sector [11], and that of rural facilities where pressures of finding substitute staff is difficult due to workforce shortages [11]. Facilities exhibiting strong cultural barriers at the organisational level reinforce the professional norms against taking sick leave, which can unwittingly encourage presenteeism [11,18,31]. Furthermore, due to the caring nature of the profession, reliance on team work, and enhanced sense of loyalty as a result of nursing socialization processes, presenteeism can also be inadvertently promoted, as nurses, who despite their sub-optimal health, do not want to let their colleagues down [15,18,32]. As noted by Rainbow [29], nurses identifying with the self-sacrifice culture promote presenteeism by reporting for shifts whilst unwell, and they also expect their colleagues to do the same.

Consistent with the report, and as highlighted in the case study analysis, the threat of COVID-19 was initially poorly understood or not taken seriously [19]. The finding that staff continued to congregate closely together in confined spaces during shift handovers, during ward rounds and attended meetings in small rooms with inadequate ventilation [19], as well as periodically consoling each other physically with hugs [19] (p. 66), supports the concept of presenteeism being a contributing factor in the transmission of COVID-19. In the later stages of the outbreak, as shown by the Haddon matrix analysis (Table 1), the physical environment proved problematic for infection control measures and was not conducive for promoting social distancing, with several co-located facilities housed within the hospital environment, such as the cafeteria and coffee shop, where individuals congregated frequently. The Haddon matrix shows the complexity of staff interactions, including high mobility across public and private health care environments because of the corridor connection. The consequences of staff casualization and the fact that health professionals worked across the differing clinical environments, including the RACFs, as often occurs in rural communities, contributed to the spread of infection [19]. The report identified that impeded best practice infection control measures contributed adversely to the severity of the North West outbreak [19,21]. The transmission process was understated and poorly understood in this rural healthcare facility, just as it was globally, in the initial phases of COVID-19 pandemic.

## 5. Limitations

The focus of the paper was presenteeism, and as such the exploration of other contributing factors that may have influenced the analysis process and the discussion may be skewed. The use of secondary data for the analysis process did not enable the seeking of further clarification of information and there is a reliance on information curated by outside parties.

## 6. Recommendations

The COVID-19 outbreak in the North West of Tasmania provides opportunities to consider strategies and recommendations which can be employed to better prepare health care environments and the healthcare workforce for infectious disease disaster management planning. Recommendations include the provision for improved infection control measures, contact tracing technologies, enhanced communication strategies including health professional education, and support for a positive work culture (Table 1). Investment of a whole-community disaster planning approach within rural areas would facilitate the mitigation of risk. Best practice disaster preparedness and management plans need to take healthcare professional presenteeism into account and address the drivers of presenteeism is a paramount consideration [9], to ensure that both staff and patients remain safe.

With specific regard to presenteeism, the review noted:

“*That the level of vigilance now required to reduce work presenteeism while suffering mild symptoms must be greatly increased to counter inadvertent transmission of COVID-19 in health care settings*.”

[19] (p. 33).

These recommendations can be equally applied across all health care environments and for any situation, including pandemics. Further research such as collecting primary research from future infectious disease outbreaks would contribute to the improvement of pandemic preparedness.

## 7. Conclusions

The complexities surrounding the first nosocomial outbreak of COVID-19 in a rural region of Australia were multifactorial. Amongst other factors, staff presenteeism was found to be a major contributor to the spread of COVID-19 across multiple healthcare settings within the rural environment. Healthcare workforce factors, coupled with social and physical environments, as shown by the Haddon matrix, contributed to the COVID-19 outbreak. Lack of disaster management planning and precarious resourcing further enabled the rapid escalation of the outbreak across the North West region of Tasmania. Healthcare workforce presenteeism compounded the situation by health professionals continuing to attend work while unwell, which posed a credible risk to colleagues, patients, and members of the community external to place of employment. Transmission of disease via workforce presenteeism behaviours can also result in localized community outbreaks, potentiating major economic and social disruption, and heightening community anxiety. This case study analysis supports the need for co-created disaster preparedness to effectively manage current healthcare professional presenteeism in rural healthcare facilities and to mitigate risks of potential community transmission during future pandemics.

## Figures and Tables

**Table 1:** Haddon matrix [22] demonstrating the complex of factors contributing to the nosocomial COVID-19 outbreak.

	**Healthcare Professional**	**Resources**	**Physical Environment**	**Social Environment**
**Pre-event** (before the outbreak occurred)	Healthcare professional presenteeism [25]	Inadequate disaster management plan or preparation for rural healthcare environments [19]	Inadequate disaster management plan or preparation for rural healthcare environments [19]; Corridor interconnection between North West public and private hospital [19]	Parochialism [19]; Casualized health care workforce [19]; Staff mobility across healthcare facilities [19]
**Event** (the nosocomial outbreak)	Healthcare professional presenteeism [25]	Fomite transmission, lack of resources including PPE and hand sanitizer [25]; Incorrect use of PPE in a range of healthcare settings [19]	Inadequate built environment, wide corridors, small meeting rooms, no negative air pressure rooms (Tasmanian Government 2020); layout features including workflows such as delivery and collection of pharmacy supplies [19]; Corridor interconnection between North West public and private hospital [19]	Lack of social distancing; fear of retribution; dismissive behaviour regarding seriousness of risk [19]; Parochialism [19]; Change in criteria of close contact during outbreak [19]; Hugging to console colleagues [19]
**Post event** (after the outbreak has been controlled)	Health care professional education and cultural change [19]	Disaster management plan, including resources available [19]	Improved contact tracing and technology to track staff and patients within and between healthcare facilities [19]	Positive work culture; improved well-being and support; anonymized staff surveys conducted [19]
